# Role of orexin in modulating arousal, feeding, and motivation

**DOI:** 10.3389/fnbeh.2013.00028

**Published:** 2013-04-18

**Authors:** Natsuko Tsujino, Takeshi Sakurai

**Affiliations:** Department of Molecular Neuroscience and Integrative Physiology, Graduate School of Medical Science, Kanazawa UniversityKanazawa, Japan

**Keywords:** orexin A, orexins, orexin receptors, hypothalamus, sleep, feeding behavior, reward

## Abstract

Orexin deficiency results in narcolepsy in humans, dogs, and rodents, suggesting that the orexin system is particularly important for maintenance of wakefulness. However, orexin neurons are “multi-tasking” neurons that regulate sleep/wake states as well as feeding behavior, emotion, and reward processes. Orexin deficiency causes abnormalities in energy homeostasis, stress-related behavior, and reward systems. Orexin excites waking-active monoaminergic and cholinergic neurons in the hypothalamus and brain stem regions to maintain a long, consolidated waking period. Orexin neurons also have reciprocal links with the hypothalamic nuclei, which regulates feeding. Moreover, the responsiveness of orexin neurons to peripheral metabolic cues suggests that these neurons have an important role as a link between energy homeostasis and vigilance states. The link between orexin and the ventral tegmental nucleus serves to motivate an animal to engage in goal-directed behavior. This review focuses on the interaction of orexin neurons with emotion, reward, and energy homeostasis systems. These connectivities are likely to be highly important to maintain proper vigilance states.

## Introduction

Neuropeptides orexin A and orexin B were originally identified as endogenous ligands for two orphan G-protein-coupled receptors (GPCRs) (Sakurai et al., 1998). They were independently identified as putative peptides encoded by a hypothalamus-specific transcript, and named hypocretin-1 and -2 (de Lecea et al., 1998). They were initially recognized as regulators of feeding behavior, because of their exclusive production in the lateral hypothalamic area (LHA), a region known as the feeding center, and owing to their pharmacological activity (Sakurai et al., 1998; Edwards et al., 1999; Haynes et al., 2000, 2002). Subsequently, the importance of orexins in the maintenance of consolidated sleep/wake states has been demonstrated by the fact that the sleep disorder narcolepsy is caused by orexin deficiency in human and animals (Chemelli et al., 1999; Lin et al., 1999; Peyron et al., 2000; Thannickal et al., 2000; Hara et al., 2001).

Recent reports also suggest that orexin is involved in emotion, stress response, and reward systems. Findings on the input and output systems of orexin neurons, as well as phenotypic characterization of mice with genetic alterations in the orexin system, suggest that orexins elicit appropriate levels of arousal to engage goal-directed behaviors by integrating the body's external and internal state, which is beneficial for survival (Yamanaka et al., 2003a; Akiyama et al., 2004; Mieda et al., 2004; Boutrel et al., 2005; Harris et al., 2005; Sakurai et al., 2005; Narita et al., 2006; Yoshida et al., 2006). This review provides an overview of the role of the orexin system especially in arousal, energy homeostasis, stress, and motivation.

### Orexin and orexin receptors

#### Identification of orexin (hypocretin)

**Figure 1:**
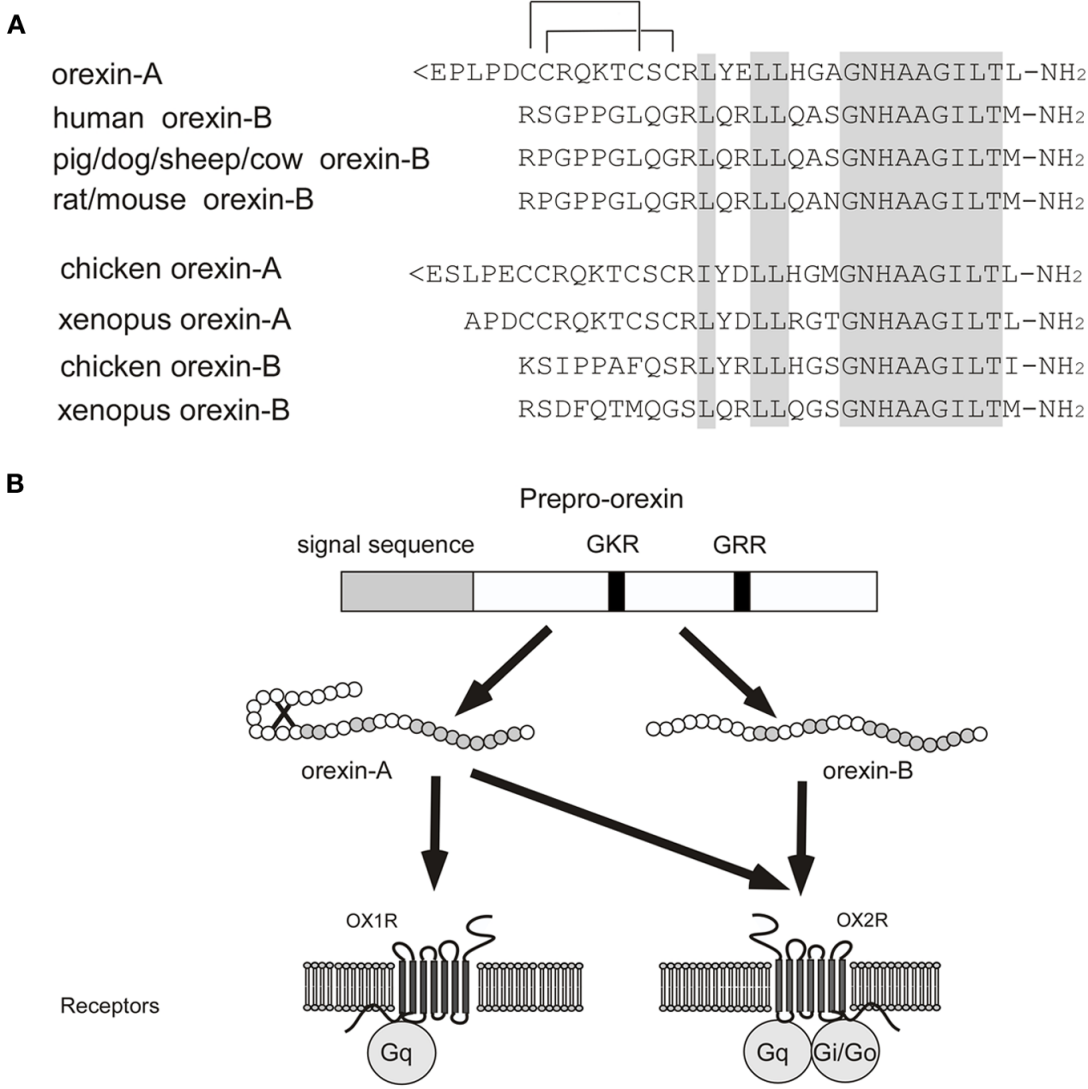
**Orexin and orexin receptors. (A)** Structure of mature orexin A and orexin B peptides. The topology of the two intrachain-bonds in orexin A is indicated above the sequence. Amino acid identities are indicated by *shading*. Mammalian orexin A sequences thus far identified (human, rat, mouse, pig, dog, sheep, and cow) are all identical, while the sequences of orexin B show some differences among species. **(B)** Schematic representation of orexin system. Orexin A and orexin B are derived from a common precursor peptide, prepro-orexin. The actions of orexins are mediated via two G protein-coupled receptors named orexin-1 (OX_1_R) and orexin-2 (OX_2_R) receptors. OX_1_R is selective for orexin A, whereas OX_2_R is a non-selective receptor for both orexin A and orexin B. OX_1_R is coupled exclusively to the G_q_ subclass of heterotrimeric G proteins, whereas OX_2_R couples to G_i/o_ and/or G_q_.

In 1998, orexin A and B were identified from rat brain extracts as ligands of an orphan GPCR, HFGAN72 (orexin receptor-1; OX_1_R) (Sakurai et al., 1998). Orexins constitute a novel peptide family with no significant structural similarities to known families of regulatory peptides. Orexin A is a 33-amino-acid peptide with two sets of intrachain disulfide bonds. It has an N-terminal pyroglutamyl residue and C-terminal amidation (Sakurai et al., 1998). The primary structure of orexin A predicted from the cDNA sequences is completely conserved among several mammalian species (human, rat, mouse, cow, sheep, dog, and pig). On the other hand, rat orexin B is a 28-amino-acid, C-terminally amidated linear peptide, which has 46% (13/28) sequence identity to orexin A. Orexin B also has a high degree of sequence similarity among species, although substantial species variants were found (Sakurai et al., 1998; Shibahara et al., 1999; Alvarez and Sutcliffe, 2002) (Figure 1A) (Shibahara et al., 1999; Alvarez and Sutcliffe, 2002; Sakurai, 2005). Orexin A and B are produced from a common single precursor polypeptide, prepro-orexin, through usual proteolytic processing presumably by prohormone convertases (Figure 1B). An mRNA encoding the same precursor peptide was independently isolated by de Lecea et al. as a hypothalamus-specific transcript. They predicted that the precursor encoded two neuropeptides, hypocretin-1 and -2 (de Lecea et al., 1998).

#### Orexin receptors

*HFGAN72*, now called *OX*_*1*_*R*, was initially identified as an expressed sequence tag (EST) from human brain (Sakurai et al., 1998). Subsequently, *orexin receptor-2* (*OX*_*2*_*R*) was identified by searching EST databases (Sakurai et al., 1998). Both receptor genes are highly conserved among species (Sakurai et al., 1998). OX_1_R has one order of magnitude greater affinity for orexin A than for orexin B. In contrast, OX_2_R has similar affinity for both orexin A and orexin B (Sakurai et al., 1998) (Figure 1B). OX_1_R is coupled to the G_q/11_ class of G proteins, which results in activation of phospholipase C (PLC) with subsequent triggering of the phosphatidylinositol cascade. OX_1_R also stimulates cAMP synthesis in primary rat astrocyte culture (Woldan-Tambor et al., 2011). OX_2_R is shown to be coupled to both G_q/11_ and inhibitory G_i_ proteins when expressed in cell lines (Zhu et al., 2003) (Figure 1B). Food deprivation was reported to exert a differential effect on coupling between orexin receptors and G proteins (Karteris et al., 2005).

#### Orexin-producing neurons

**Figure 2:**
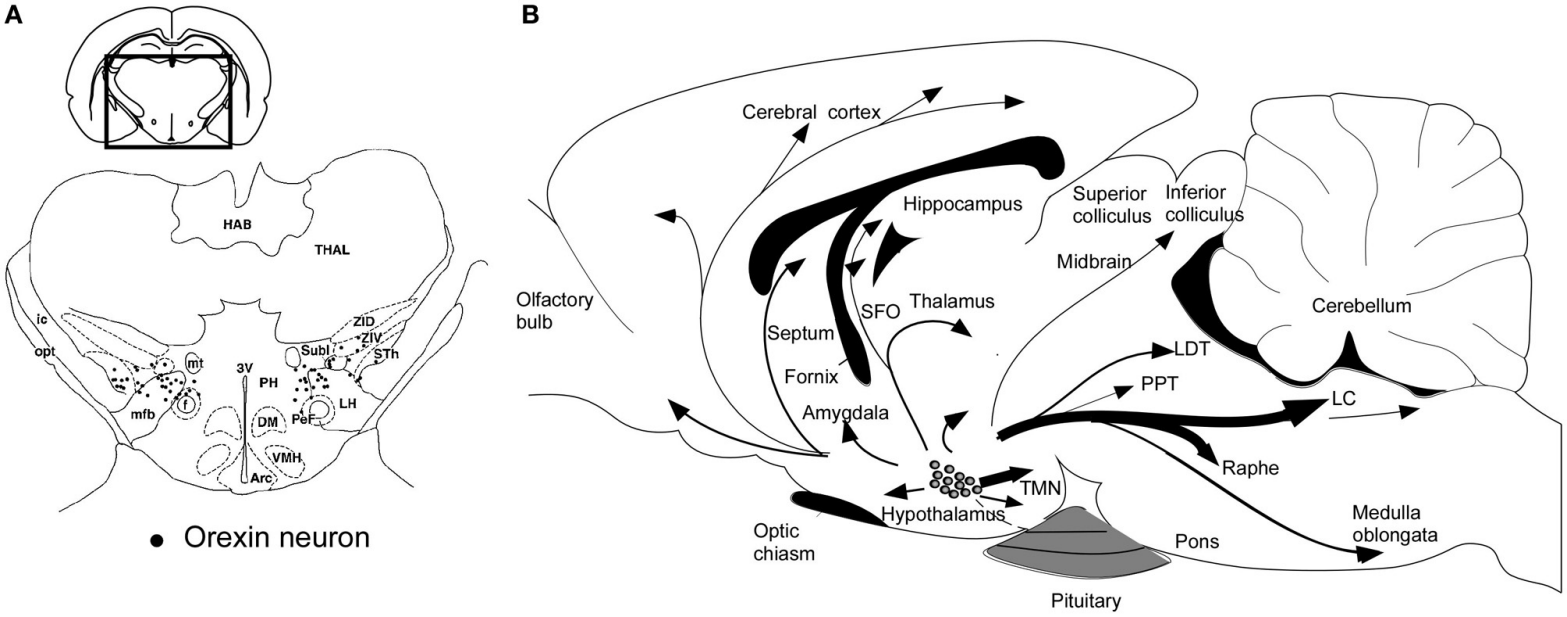
**Schematic drawing of coronal section and sagittal section through rat brain, summarizing the orexin neuronal system. (A)** *Prepro-orexin* mRNA-containing neurons are shown in black superimposed upon anatomical structures of the hypo- and subthalamic areas. The rectangle designates the area schematized in the figure. Abbreviations: LH, lateral hypothalamic area; PeF, perifornical nucleus; PH, posterior hypothalamic area; Sth, subthalamic nucleus; SubI, subincertal nucleus; ZIV, ventral zona incerta. Additional landmarks include: THAL, thalamus; HAB, habenular complex; ic, internal capsule; opt, optic tract; mt, mammillothalamic tract; f, fornix; mfb, medial forebrain bundle; 3V, third ventricle; Arc, arcuate hypothalamic nucleus; DM, dorsomedial hypothalamic nucleus; and VMH, ventromedial hypothalamic nucleus. **(B)** Orexin neurons are found only in the lateral hypothalamic area and project to the entire central nervous system. The thickness of arrows represents relative abundance of projections. Abbreviations: 3V, third ventricle; 4V, fourth ventricular; TMN, tuberomammillary nucleus; LC, locus coeruleus; LDT, laterodorsal tegmental nucleus; PPT, pedunculopontine nucleus.

Orexin-producing neurons (orexin neurons) are exclusively localized to the perifornical area and lateral and posterior hypothalamic area in the rat brain (Peyron et al., 1998; Date et al., 1999; Nambu et al., 1999) (Figure 2A). This distribution has been confirmed in human (Elias et al., 1998). The number of orexin neurons is assumed to be around 3000 in the rat brain, and 70,000 in the human brain (Peyron et al., 1998; Thannickal et al., 2000), and these cells diffusely project to the entire neuroaxis (Peyron et al., 1998; Date et al., 1999; Nambu et al., 1999) (Figure 2B). This anatomical structure suggests that the activity of orexin neurons influences multiple brain areas. The strongest staining of orexin-immunoreactive nerve endings in the brain was found in the tuberomammillary nucleus (TMN), paraventricular thalamic nucleus (PVT), arcuate nucleus (Arc) of the hypothalamus, and most notably, monoaminergic nuclei in the brain stem, such as the locus ceruleus (LC) which receives the densest oreinergic fibers in the brain stem, and raphe nuclei.

Orexin colocalizes with dynorphin (Chou et al., 2001), galanin (Hakansson et al., 1999), prolactin (Risold et al., 1999), neuronal activity-regulated pentraxin (NARP) (Reti et al., 2002), glutamate (Abrahamson et al., 2001), and neurotensin (unpublished data). Many orexin neurons are glutamatergic (Rosin et al., 2003; Torrealba et al., 2003) but are not GABAergic (Rosin et al., 2003). A recent study using an optogenetic technique confirmed that orexin neurons released glutamate on TMN histaminergic neurons (Schone et al., 2012).

#### Distribution of orexin receptors

In the central nervous system, both orexin receptor mRNAs are expressed in regions that receive dense orexin innervations as described above. *OX*_*1*_*R* and *OX*_*2*_*R* mRNAs show partial overlap, but largely distinct and complementary distribution patterns, suggesting that each receptor subtype plays different physiological roles. *OX*_*1*_*R* is expressed in many brain regions such as the prefrontal and infralimbic cortex (IL), hippocampus (CA2), amygdala, bed nucleus of the stria terminalis (BST), PVT, anterior hypothalamus, dorsal raphe (DR), ventral tegmental area (VTA), LC, and laterodorsal tegmental nucleus (LDT)/pedunculopontine nucleus (PPT) (Trivedi et al., 1998; Lu et al., 2000; Marcus et al., 2001). *OX*_*2*_*R* is expressed in the amygdala, TMN, Arc, dorsomedial hypothalamic nucleus (DMH), paraventricular nucleus (PVN), LHA, BST, PVT, DR, VTA, LDT/PPT, CA3 in the hippocampus, and medial septal nucleus (Lu et al., 2000; Marcus et al., 2001). Double *in situ* hybridization studies have revealed the expression patterns of *OX*_*2*_*R* and *OX*_*1*_*R* mRNA more precisely. In the TMN, all *vesicular monoamine transporter 2 (VMAT2)*-positive histaminergic neurons expressed *OX*_*2*_*R* mRNA, while *OX*_*1*_*R* mRNA was not detected. In the DR and median raphe nuclei (MnR), the majority of *VMAT2-*positive serotonergic neurons expressed both *OX*_*1*_*R* and *OX*_*2*_*R* mRNA. In the LC, all *VMAT2*-positive noradrenergic neurons exhibited *OX*_*1*_*R*, whereas *OX*_*2*_*R* mRNA was exclusively detected in *VMAT2*-negative non-noradrenergic neurons. In the LDT and PPT, all *vesicular acetylcholine transporter* (*VAChT*)-positive cholinergic neurons expressed *OX*_*1*_*R* but not *OX*_*2*_*R* mRNA, but many *OX*_*1*_*R*-positive and/or *OX*_*2*_*R*-positive noncholinergic neurons were intermingled with cholinergic neurons (Mieda et al., 2011). These histological findings suggest that orexins and their receptors are likely to play a broad regulatory role in the monoaminergic/cholinergic systems.

### Input and output of orexin neurons

#### Neuronal afferents

In mice with a genetically encoded retrograde tracer and in rats with combined antero- and retrograde tracers, upstream neuronal populations that make innervations to orexin neurons were revealed (Sakurai et al., 2005; Yoshida et al., 2006). These studies showed that orexin neurons are innervated by the lateral parabrachial nucleus (LPB), ventrolateral preoptic nucleus (VLPO), medial and lateral preoptic areas, basal forebrain (BF), posterior/dorsomedial hypothalamus, VTA, DR nucleus, and MnR. Many upstream neurons were identified in regions associated with emotion including the IL, amygdala, shell region of the nucleus accumbens (NAc), lateral septum (LS) and BST. Orexin neurons were also shown to receive innervations from regions associated with energy homeostasis including NPY-, agouti-related peptide (AgRP)-, and α-melanin-stimulating hormone-immunoreactive fibers, which presumably originate in the arcuate nucleus (Broberger et al., 1998; Elias et al., 1998).

#### Factors that influence activity of orexin neurons

A number of factors that influence firing rate or membrane potential of orexin neurons have been identified (Table 1).

**Table 1:** **Factors that influence activity of orexin neurons**.

**Excitation**	**Receptor involved**
Glutamate	AMPAR, NMDAR mGluRs
Acetylcholine (muscarinic) (27%)	M3
Orexin	OX_2_R
Ghrelin	GHSR
Cholecystokinin	CCKA
Neurotensin	NTSR2 (unpublished data)
Vasopressin	V1a
Oxytocin	V1a
Glucagon-like peptide 1	ND
Corticotropin-releasing factor	CRFR1
Thyrotropin-releasing hormone	TRH1
BRS3 agonist	BRS3
ATP	P2X
H^+^	ASIC1a
CO_2_	ND
Mixture of amino acids	System-A amino acid transporters
**INHIBITION**	
Glucose	Unknown
GABA	GABA_A_, GABA_B_
Glycine	Glycine receptor
Serotonin	5HT_1A_
Noradrenaline	α_2_
Dopamine	α_2_
Acetylcholine (muscarinic) (6%)	ND
Neuropeptide Y	Y_1_
Enkephalin	μ opioid-R
Nociceptin	NOPR
Leptin	OBR
Adenosine	A_1_
BRS3 agonist (indirect)	BRS3

Importantly, both noradrenaline and serotonin (5HT), both of which are wake-active substances, hyperpolarize and inhibit activity of orexin neurons through activation of G protein-regulated inwardly rectifying K^+^ channels via α_2_-adrenoceptors and 5HT_1A_-receptors, respectively (Li et al., 2002; Muraki et al., 2004; Yamanaka et al., 2003b, 2006).

In addition, the cholinergic agonist carbachol activates some orexin neurons through M3 muscarinic receptors (Yamanaka et al., 2003b; Sakurai et al., 2005). However, histamine appeared to have no effect on orexin neurons. These observations suggest that serotonin and noradrenaline neurons might send inhibitory feedback projections to orexin neurons. These feedback mechanisms might stabilize the activity of both orexin neurons and monoaminergic neurons. Furthermore, although orexin neurons do not express functional dopamine receptors, dopamine can inhibit orexin neurons by acting on α_2_-adrenoceptors (Yamanaka et al., 2003b, 2006). It was also shown that agonists of ionotropic glutamate receptors excite orexin neurons, whereas glutamate antagonists (AP-5, CNQX, or NBQX) reduce their activity (Li et al., 2002; Yamanaka et al., 2003b). These results indicate that orexin neurons are tonically excited by glutamatergic neurons. At the same time, GABAergic and glycinergic input to orexin neurons strongly inhibits the activity of orexin neurons (Xie et al., 2006; Matsuki et al., 2009; Hondo et al., 2011; Karnani et al., 2011b).

In addition, a sulfated octapeptide form of cholecystokinin (CCK-8S), neurotensin, oxytocin, vasopressin, orexin, and a mixture of amino acids activate orexin neurons (Tsujino et al., 2005; Tsunematsu et al., 2008; Yamanaka et al., 2010; Karnani et al., 2011a), whereas glucose and leptin inhibit them (Table 1). These factors are implicated in energy homeostasis. It was also shown that adenosine inhibits orexin neurons via the A_1_ receptor. This pathway might be related to the sleep-promoting effect of adenosine (Liu and Gao, 2007). In addition, orexin neurons are affected by physiological fluctuations in acid and CO_2_ level (Williams et al., 2007). Since orexin affects respiratory function, this mechanism might play a role in the regulation of respiratory function (Nakamura et al., 2007; Sunanaga et al., 2009). A recent report suggested that acid-sensing ion channels 1a (ASIC1a) located on orexin neurons contribute to the regulation of breathing by sensing local acidity (Song et al., 2012). These results suggest that orexin neurons are specialized sensors of the internal environment.

### Orexin deficiency causes narcolepsy

Narcolepsy is a sleep disorder characterized by a primary disorganization of behavioral states. This disorder affects ~1 in 2000 individuals in the United States (Mignot, 1998). Most cases of human narcolepsy start during adolescence and persist throughout life. Many experiments have revealed that human narcolepsy is caused by orexin deficiency (Chemelli et al., 1999; Lin et al., 1999; Peyron et al., 2000; Thannickal et al., 2000). Narcolepsy is characterized by the inability to maintain vigilance states, pathological intrusion of non-rapid eye movement (NREM) and/or rapid eye movement (REM) sleep into wakefulness, and frequent transitions between states of sleep and wakefulness. Human narcolepsy patients experience excessive daytime sleepiness, manifested particularly as attacks of falling asleep at inappropriate times. They often suffer attacks of sudden weakening of postural muscle tone, called “cataplexy.” These attacks are often triggered by emotional stimuli.

The first clues to the involvement of the orexins in narcolepsy came from animal models. *Prepro-orexin* gene knockout mice or dogs with null mutations in the *OX*_*2*_*R* gene show phenotypes remarkably similar to humans with narcolepsy (Chemelli et al., 1999; Lin et al., 1999). *Prepro-orexin* knockout mice, orexin neuron-ablated (*orexin/ataxin-3*-transgenic) mice, and *OX_1_R/OX_2_R* double knockout mice showed highly similar phenotypes to the human condition, characterized by behavioral arrests similar to cataplexy, occasional direct transitions to REM sleep from wakefulness, and highly fragmented sleep-wake cycles (Chemelli et al., 1999; Hara et al., 2001; Mochizuki et al., 2004). In zebrafish, ablation of orexin neurons also increased sleep time and the number of sleep/wake transitions. This report also suggested that ablation of orexin neurons altered behavioral state transitions in response to external inputs (Elbaz et al., 2012).

Consistently, postmortem studies of human narcolepsy subjects showed an 80–100% reduction in the number of neurons containing detectable *prepro-orexin* mRNA or orexin-like immunoreactivity in the hypothalamus and undetectable levels of orexin peptides in projection sites (Figures 2A,B) (Peyron et al., 2000; Thannickal et al., 2000). In good agreement with this report, an undetectable level of orexin A in the cerebrospinal fluid (CSF) of narcolepsy patients has been reported (Nishino et al., 2000; Mignot et al., 2002). A low CSF level of orexin A is now one of the diagnostic criteria for narcolepsy-cataplexy (American Academy of Sleep Medicine, 2005).

Furthermore, a concomitant loss of dynorphin, NARP, and orexin, which colocalize in orexin neurons, suggests a loss of orexin neurons in narcolepsy-cataplexy (Crocker et al., 2005). The cause of the specific loss of orexin neurons in narcolepsy is unknown thus far, but because of its strong association with certain HLA alleles (Kadotani et al., 1998) it is possible that narcolepsy may be an autoimmune disorder. Recently, the transcript encoding Tribbles homolog 2 (Trib2), an autoantigen in autoimmune uveitis, was reported to be enriched in orexin neurons, and has been considered as a possible candidate antigen responsible for the autoimmunity. Serum from narcolepsy patients had higher Trib2-specific antibody titers compared with normal controls (Murillo-Rodriguez et al., 2008).

Because narcolepsy is a disorder resulting from an absence of orexin, replacement therapy may provide an effective treatment for narcolepsy. In fact, acute administration of orexin A or chronic overproduction of orexin from an ectopically expressed transgene prevented cataplectic arrests and other abnormalities of REM sleep in orexin neuron-ablated mice (Mieda et al., 2004). Furthermore, following viral vector transfer of the gene for mouse *prepro-orexin* to the LHA of orexin-deficient mice, the incidence of cataplexy declined and abnormalities of REM sleep were improved (Liu et al., 2008). Moreover, orexin gene delivery into neurons of the zona incerta blocked cataplexy in orexin neuron-deficient mice (Liu et al., 2011). Orexin gene transfer into the dorsolateral pons significantly decreased cataplexy and modestly improved wake maintenance (Blanco-Centurion et al., 2013). These results indicate that narcoleptic mice retain the ability to respond to orexin and that temporally regulated and spatially targeted secretion of orexins is not necessary to prevent narcoleptic symptoms.

The deficiency of orexin signaling in narcolepsy symptoms and pathophysiology suggests that orexins play important roles in the regulation of sleep and wakefulness, especially in their stabilization, as well as inhibition of REM sleep and REM sleep-related phenomena.

### Roles of orexins in regulation of sleep/wake states

#### Interactions with sleep and waking centers

**Figure 3:**
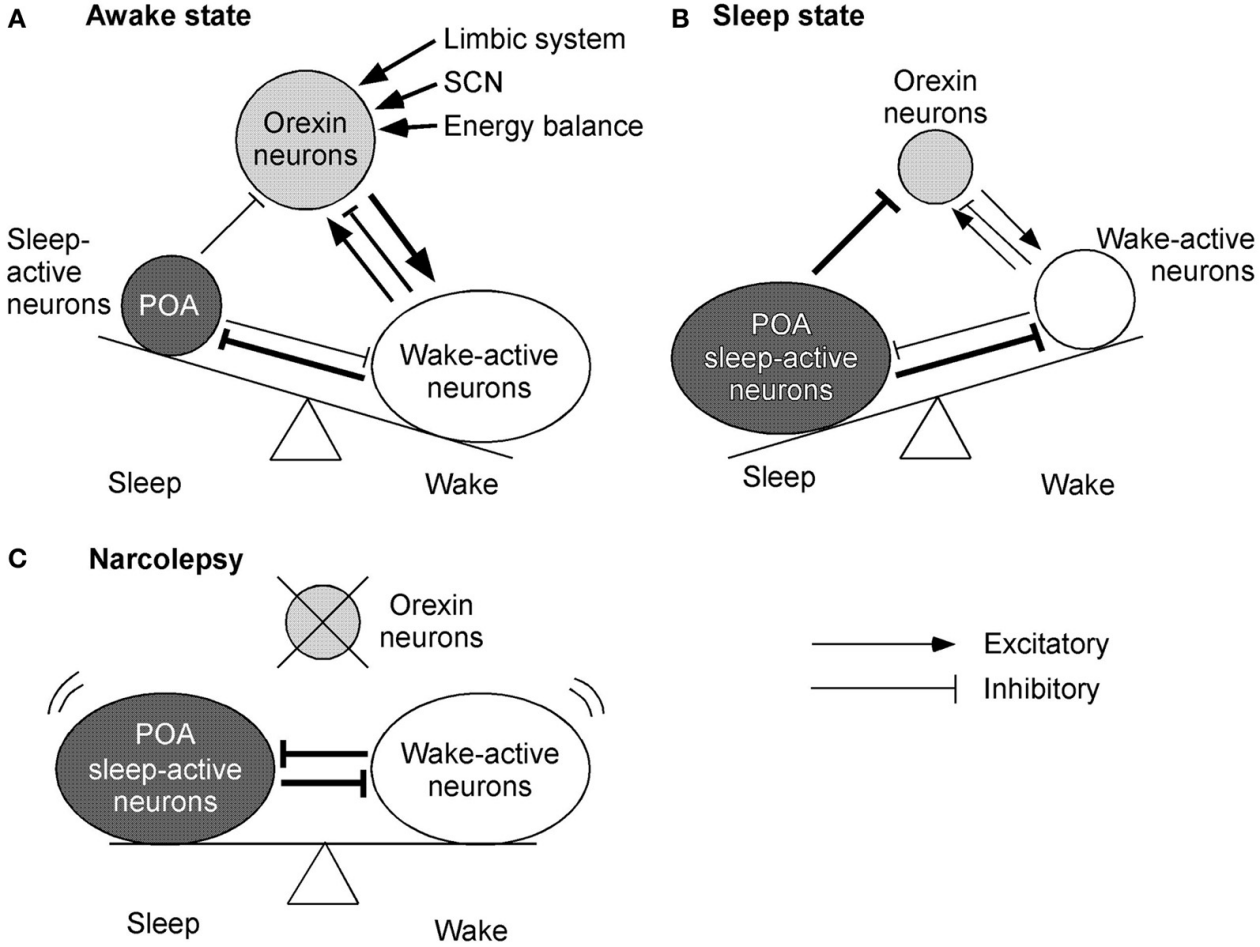
**Mechanisms by which orexin system maintains consolidated sleep and wakefulness.** The figures represent the functional interaction between orexin neurons, wake-active centers, and sleep-active centers during various states of sleep and wakefulness. Arrows show excitatory and lines show inhibitory input. The thickness of arrows and lines represent relative strength of input. Circle sizes represent relative activities of each group of neurons **(A)** Awake state. Orexin neurons send excitatory input to wake-active neurons, which send inhibitory feedback projections to orexin neurons. This system might maintain the activity of wake-active neurons. A small decrease in the activity of wake-active neurons results in decreased inhibitory influence to orexin neurons. Orexin neurons, therefore, are disinhibited and increase their excitatory influence on wake-active neurons to maintain their activity. These wake-active neurons send inhibitory projections to the POA sleep center and excitatory projections to the thalamus and cerebral cortex. **(B)** Sleep state. GABAergic neurons in the sleep center are activated and send inhibitory projections to wake-active neurons and orexin neurons to maintain a sleep state. **(C)** Model of narcolepsy. Sleep-active neurons in the POA inhibit wake-active neurons and in turn are inhibited by them, thus forming a mutually inhibitory system. This system can cause unnecessary transition between the states, because when either side begins to overcome the other, the switch abruptly turns into the alternative state.

Sleep-active neurons in the POA, especially the ventrolateral preoptic area (VLPO), appear to play a critical role in the initiation of NREM sleep and maintenance of both NREM and REM sleep (Sherin et al., 1998). Neurons in the VLPO fire at a rapid rate during sleep, with attenuation of firing during the waking period (Figures 3A,B). These neurons mostly contain GABA and/or galanin, and send descending inhibitory projections to wake-active neurons that produce wake-promoting neurotransmitters, including histamine, noradrenaline, 5-HT, and acetylcholine (Sherin et al., 1998; Lu et al., 2002) (Figures 3A,B). On the contrary, these sleep-promoting neurons are inhibited by wake-active transmitters such as noradrenaline, acetylcholine, and 5-HT (Gallopin et al., 2000). These reciprocal interactions of inhibition constitute the flip-flop switching of wake/sleep states (Saper et al., 2001). Importantly, GABAergic neurons in the POA also densely innervate orexin neurons (Sakurai et al., 2005; Yoshida et al., 2006). This pathway might be important to turn-off orexin neurons during sleep (Figure 3B). In fact, orexin neurons are strongly inhibited by both GABA_A_ and GABA_B_ receptor agonists (Yamanaka et al., 2003a; Xie et al., 2006). Moreover, selective deletion of the GABA_B_ receptor gene in orexin neurons resulted in highly unstable sleep/wake architecture in mice (Matsuki et al., 2009).

As already mentioned, narcolepsy patients and animals with defects of orexin or OX2R cannot maintain a consolidated wakefulness state. It is recognized that monoaminergic neurons in the hypothalamus and brain stem, including neurons in the TMN, LC, and DR, play crucial roles in maintaining arousal (Saper et al., 2005). Firing rates of these neurons are known to be synchronized and strongly associated with sleep/wake states. They fire tonically during the awake state, less during NREM sleep, and are virtually quiescent during REM sleep (Vanni-Mercier et al., 1984). Orexin neurons were also reported to discharge during active wakefulness and cease firing during both NREM and REM sleep (Lee et al., 2005), or displayed transient discharge in REM, *in vivo* (Gerashchenko and Shiromani, 2004; Blanco-Centurion et al., 2007). Furthermore, many studies also suggested that noradrenergic cells of the LC (Hagan et al., 1999; Bourgin et al., 2000), dopaminergic cells of the VTA (Nakamura et al., 2000), serotonergic cells of the DR (Brown et al., 2002; Liu et al., 2002), histaminergic cells of the TMN (Yamanaka et al., 2002), and cholinergic neurons in the BF (Eggermann et al., 2001) are all excited by orexins *in vitro* (Alam et al., 1999). These results suggest that orexin neurons fire during the wakeful period, and excite these wake-active neurons to sustain their activity.

On the other hand, as described previously, orexin neurons are regulated by acetylcholine and monoamine. Constantly, orexin neurons are innervated by BF-cholinergic neurons (Sakurai et al., 2005). A cholinergic agonist, carbachol, activates some populations of orexin neurons. Furthermore, serotonergic and noradrenergic neurons send inhibitory projections to orexin neurons (Muraki et al., 2004; Sakurai et al., 2005; Yamanaka et al., 2006). These findings indicate that orexin neuron maintain an awake state by activation of wake-active neurons. On the other hand, during a sleep state, sleep-active neurons inhibit both orexin neuron and wake-active neurons to maintain a sleep state. If orexin neurons were deleted as in narcolepsy, sleep-active neurons and wake-active neurons would make a mutually inhibitory system. This might result in behavioral instability, which is a major symptom of narcolepsy (Figure 3C).

Additionally, optogenetic activation of orexin neurons expressing channelrhodopsin-2 increased the probability of transition to wakefulness from sleep (Adamantidis et al., 2007). This effect was observed throughout the entire light/dark period, but was diminished with sleep pressure (Carter et al., 2009). Carter et al. suggested that the orexin system promotes wakefulness throughout the light/dark period, but the downstream targets are inhibited with increased sleep pressure overriding the effect of orexin. Optogenetic inhibition of orexin neurons expressing halorhodopsin resulted in induction of slow wave sleep during the light period, although it had no effect during the dark period (Tsunematsu et al., 2011). Pharmacogenetic modulation of orexin neurons also revealed that excitation of orexin neurons increased wakefulness time and inhibition of orexin neurons decreased wakefulness time and increased NREM sleep (Sasaki et al., 2011). These selective modulations of orexin neurons *in vivo* suggest that activity of orexin neurons actually influences an animal's vigilance states. Furthermore, optogenetic modulation of orexin neurons and LC neurons revealed that the wake-promoting influence of orexin is mediated by LC neurons (Carter et al., 2012).

#### Contribution of OX_1_R and OX_2_R in regulation of wakefulness

Intracerebroventricular (icv) injection of orexin during the light period potently increases the awake period in rats, and this effect is markedly attenuated by a histamine H_1_ antagonist (Yamanaka et al., 2002). The pharmacological effect of orexin A on waking time in mice is almost completely absent in H_1_-receptor-deficient mice (Huang et al., 2001). Moreover, focal restoration of OX_2_R in neurons of the TMN and adjacent parts of the posterior hypothalamus in mice lacking OX_2_R completely rescued the sleepiness of these mice (Mochizuki et al., 2011). These results suggest that the TMN-histaminergic pathway might be an important effector site of orexin for sleep/wake regulation.

Consistently, *OX*_*2*_*R* knockout mice show characteristics of narcolepsy (Willie et al., 2003), while *OX*_*1*_*R* knockout mice exhibit only very mild fragmentation of the sleep-wake cycle (Willie et al., 2001). However, notably, the phenotype of *OX*_*2*_*R* knockout mice is far less severe than that found in *prepro-orexin* knockout mice and double receptor knockout mice. Especially, *OX*_*2*_*R* knockout mice are only mildly affected by cataplexy and direct transitions to REM sleep from an awake state. Furthermore, the effects of orexin A on wakefulness and NREM sleep were significantly attenuated in OX_2_R knock-out mice as compared with wild-type mice, but OX_1_R knockout mice also showed an impaired response (Mieda et al., 2011). These observations suggest that although the OX_2_R-mediated pathway has a pivotal role in the promotion of wakefulness, OX_1_R also has additional effects on sleep-wake regulation. These findings suggest that loss of signaling through both OX_1_R- and OX_2_R-dependent pathways is necessary for emergence of a complete narcoleptic phenotype.

#### REM-related atonia

Cataplexy has been proposed to be controlled by mechanisms similar to those producing atonia during REM sleep. Recent reports suggested that the REM-related atonia is induced by activation of REM-on neurons in the sublaterodorsal tegmental nucleus (SLD) (Fuller et al., 2007; Luppi et al., 2011). One of the activation pathways of REM-on neurons is likely to be mediated by input from the amygdala, which is well-known to be activated during REM sleep (Luppi et al., 2011). Strong, generally positive emotional stimuli, which might activate the amygdala, are known to trigger cataplexy in narcolepsy-cataplexy patients. These suggest that the amygdala-induced REM-on neuron activation might play a role in cataplexy. During wakefulness and NREM sleep, the SLD REM-on neurons are inhibited by REM-off neurons in the ventrolateral periaquaductal gray and dorsal deep mesencephalic reticular nucleus (vlPAG/dDPMe) (Luppi et al., 2011). These findings suggest a possibility that excitatory input from orexin neurons to vlPAG/dDPMe supports the activity of these REM-off neurons to avoid muscle atonia. This hypothesis explains why narcoleptic patients exhibit cataplexy during emotional events. Moreover, neural input from the limbic system, especially the amygdala, to orexin neurons might also be implicated in the pathophysiology of cataplexy. Some report also suggested that cholinergic neurons in the LDT/PPT are implicated in REM-related atonia (Shiromani et al., 1988), and the same pathway is implicated in cataplexy. A local injection of orexin into the PPT strongly inhibited REM-related atonia in cats (Takakusaki et al., 2005). It is possible to speculate that emotional stimuli may increase orexin release in the PPT, which indirectly inhibits cholinergic neurons, to prevent muscle atonia in wild-type animals. Another report also showed that orexin activates lateral vestibular nucleus neurons and promotes vestibular-mediated motor behavior. This report suggests that orexin is critical when an animal is facing a major motor challenge. The orexin system participates not only in sleep and emotion but also in motor regulation directly. These findings may also account for the mechanism of cataplexy (Zhang et al., 2011).

It is known that food perception often evokes cataplexy in narcoleptic dogs and orexin knockout mice (Reid et al., 1998; Clark et al., 2009), suggesting that orexin signaling is physiologically activated upon perception of food, and that this system is necessary to evoke proper feeding behavior. This suggests that regulation of feeding behavior might also be controlled by input to orexin neurons from the limbic system, because some of the affective content of the perception of food is thought to be processed in the amygdala and limbic system (Berthoud, 2004), and this information may be passed on to orexin neurons.

### Role of orexins in stress response

Orexin neurons are activated by a variety of emotional and physical stressors including a resident intruder paradigm, air-jet stress, cold exposure, food deprivation, foot shock, and immobilization stress (Sakurai et al., 1998; Ida et al., 2000; Salin-Pascual et al., 2001). Orexin administration promotes a variety of autonomic responses associated with a stress state, including elevation of blood pressure, heart rate, oxygen consumption, body temperature, energy metabolism, and respiration (Lubkin and Stricker-Krongrad, 1998; Samson et al., 1999; Shirasaka et al., 1999; Yoshimichi et al., 2001; Shahid et al., 2011; Tupone et al., 2011). Administration of orexin also elevated plasma corticosterone level (Hagan et al., 1999). A recent study also suggested that activation of orexin neurons is necessary for developing a panic-prone state in a rat panic model. Moreover, human subjects with panic anxiety have elevated levels of orexin in the CSF (Johnson et al., 2010). These findings suggest that input coming from regions implicated in emotion and stress might be important for regulation of the orexin system, and activation of orexin neurons might induce an increase in sympathetic outflow and stress response.

Orexin neurons receive input from the limbic system (Winsky-Sommerer et al., 2004; Sakurai et al., 2005; Yoshida et al., 2006), and the importance of this connection is shown in the defense, or “fight or flight,” response. A resident-intruder paradigm or air-jet stress-induced increases in blood pressure, heart rate, and locomotor activity were smaller in orexin-deficient mice than in wild-type mice (Kayaba et al., 2003; Zhang et al., 2006). Stimulation of the amygdala or the BST, both of which are implicated in stress-induced autonomic responses, induced cardiorespiratory excitation in wild-type but not in orexin neuron-ablated mice (Kuwaki, 2011). In addition, stress-induced analgesia (SIA) induced by foot shock was attenuated in orexin knockout mice (Watanabe et al., 2005). These results indicate that orexin neurons might be important modulators required for orchestrating the neural circuits controlling emotional behavior and autonomic functions, and act as a master switch to activate various efferent pathways of emotional responses.

Corticotrophin-releasing factor (CRF) neurons in the amygdala and the PVN receive orexin-containing fibers (Winsky-Sommerer et al., 2004). On the other hand, CRF-immunoreactive fibers are abundant in the LHA, and CRF excites orexin neurons via the CRF-R1 receptor (Winsky-Sommerer et al., 2004). The reciprocal link between the CRF system and orexin neurons might maintain wakefulness during stressful events. Indeed, activation of orexin neurons by foot shock stress is severely impaired in *CRF-R1 receptor*-deficient mice, suggesting that such activation is mediated by CRF (Winsky-Sommerer et al., 2004). On the other hand, orexin activates CRF-containing neurons, resulting in activation of the HPA axis (Kuru et al., 2000; Fuller et al., 2007).

Excessive activation of orexin neurons during the rest period by limbic input might contribute to sleep disruption under stressful conditions.

### Roles of orexins in feeding behavior and energy homeostasis

It is has been thought that the LHA is involved in food intake and energy homeostasis. Icv injection of orexins during the light period induces feeding behavior in rodents and zebrafishes (Sakurai et al., 1998; Edwards et al., 1999; Haynes et al., 2000, 2002; Yokobori et al., 2011). These observations suggest that orexin neurons might regulate feeding behavior in various species. As previously mentioned, orexin neurons are able to monitor humoral and neural indicators of energy balance. A high extracellular concentration of glucose and leptin induces marked hyperpolarization of orexin neurons. Conversely, decreased concentration of glucose, ghrelin, and a mixture of amino acids induces depolarization (Yamanaka et al., 2003a; Burdakov et al., 2005; Gonzalez et al., 2009; Schone et al., 2011). The LHA contains glucose-sensitive neurons that are activated by glucopenia and are thus implicated in the positive short-term regulation of feeding and energy expenditure (Oomura et al., 1974). The responses of orexin neurons to glucose are divided into transient and sustained inhibitory responses. These mechanisms maintain orexin neurons' response to glucose fluctuations (Williams et al., 2008). These studies suggest that orexin neurons are glucose sensitive and may play an important role in feeding and energy expenditure. Importantly, this mechanism is sufficiently sensitive to encode variations in glucose levels reflecting those occurring physiologically between normal meals (Burdakov et al., 2005). *Prepro-orexin* mRNA level is also increased in hypoglycemic conditions or after a 48 h fast, suggesting that expression of the gene is also regulated by plasma glucose level (Sakurai et al., 1998; Griffond et al., 1999; Moriguchi et al., 1999; Yamanaka et al., 2003a). These findings suggest that orexin neurons monitor indicators of energy balance of the body and mediate adaptive augmentation of arousal in response to fasting. POMC neurons and NPY neurons in the Arc have been shown to innervate orexin neurons (Elias et al., 1998). Injection of agouti-related protein (Agrp), which is an endogenous antagonist of MC3/4Rs, resulted in activation of orexin neurons (Zheng et al., 2002). These findings suggest that the orexin system is involved in the hypothalamic neuronal network that regulates feeding behavior and energy homeostasis.

The altered energy homeostasis in human narcolepsy patients also suggests roles of orexin in regulation of energy homeostasis (Honda et al., 1986; Schuld et al., 2000). The finding of decreased caloric intake (Lammers et al., 1996) combined with an increased body mass index (Schuld et al., 2000) suggests that narcolepsy patients have a feeding abnormality with reduced energy expenditure. Consistently, orexin neuron-ablated mice show hypophagia and late-onset obesity (Hara et al., 2001). One of the reasons for obesity in narcolepsy is related to impaired thermogenesis (Tupone et al., 2011). Inability of brown preadipocytes to differentiate was also observed in orexin knockout mice (Sellayah et al., 2011). Orexin also regulated muscle glucose metabolism by activating muscle sympathetic nerves and β2-adrenergic signaling (Shiuchi et al., 2009). These studies indicate that orexin regulates not only feeding behavior but also peripheral energy expenditure.

Administration of an OX_2_R selective agonist-suppressed weight gain in mice on a high-fat diet (Funato et al., 2009). Central administration of anti-orexin antibody or an OX_1_R-selective antagonist-reduced food intake (Haynes et al., 2000; Yamada et al., 2000), and *prepro-orexin* knockout mice also showed hypophagia (Willie et al., 2001). Moreover, an OX_1_R-selective antagonist-reduced food intake and ameliorated obesity in leptin-deficient *ob/ob* mice (Haynes et al., 2002), suggesting that leptin deficiency at least partly activates the orexin pathway to increase food intake. This is consistent with findings that leptin inhibited the activity of orexin neurons. The Arc receives dense projections from orexin neurons (Peyron et al., 1998; Date et al., 1999; Yamanaka et al., 2000), and *Fos* expression was induced in Arc NPY neurons by icv injection of orexin (Yamanaka et al., 2000). Electrophysiological studies showed that orexin activated NPY neurons (van den Top et al., 2004; Li and van den Pol, 2006) and inhibited proopiomelanocortin (POMC) neurons (Muroya et al., 2004; Ma et al., 2007). Furthermore, the orexin A-induced increase in food intake was partly inhibited by administration of an NPY-Y1 receptor antagonist (Yamanaka et al., 2000). These experiments suggest that orexin-stimulated food intake is at least partially mediated by activation of NPY neurons.

Infusion of orexin A or a GABA_A_ receptor agonist into the shell of the NAc increased feeding behavior (Thorpe and Kotz, 2005) and increased *Fos* expression in orexin neurons (Baldo et al., 2004). These findings indicate that projections from the NAc to orexin neurons might play a role in regulating feeding behavior.

Orexin-mediated maintenance of consolidated wakefulness states might also be important in supporting motivated behavior related to food intake, such as food seeking, because proper maintenance of arousal during food searching and intake is essential for an animal's survival. For example, when faced with reduced food availability, animals adapt with a longer awake period, which disrupts the normal circadian pattern of activity (Itoh et al., 1990; Challet et al., 1997). During starvation, orexin neurons might be activated by low leptin and glucose levels, along with a high ghrelin level. These mechanisms may directly modulate the activity of orexin neurons according to appetite and body energy stores to maintain wakefulness. Consistently, orexin neuron-ablated mice fail to respond to fasting with increased wakefulness and activity (Yamanaka et al., 2003a). These findings suggest that orexin neurons have a critical role in the maintenance of arousal during a period of negative energy balance. These properties might allow orexin neurons to promote alertness in a hungry animal and maintain long periods of wakefulness. These findings indicate that orexin neurons provide a crucial link between energy balance and arousal.

### Roles of orexins in reward systems

#### Interaction with reward system

The LHA has been implicated in the reward system by both lesion experiments and the intracranical self-stimulation paradigm (Anand and Brobeck, 1951; Olds and Milner, 1954). Recently, several lines of evidence have suggested that orexins are involved in modulation of the reward system.

Narcoleptic patients are often treated with highly addictive amphetamine-like drugs (Nishino and Mignot, 1997) but they rarely become addicted to these drugs (Akimoto et al., 1960; Guilleminault et al., 1974). Consistently, orexin knockout mice are less susceptible than wild-type mice to developing morphine dependence as measured by physical withdrawal response (Georgescu et al., 2003; Narita et al., 2006). Moreover, abnormal activity in reward brain circuits was observed in human narcolepsy (Ponz et al., 2010).

These observations suggest that orexin neurons play important roles in reward processing. In fact, VTA dopaminergic neurons receive input from orexin neurons composed of both synaptic terminals (Peyron et al., 1998; Marcus et al., 2001; Fadel and Deutch, 2002) and en-passant fibers (Balcita-Pedicino and Sesack, 2007), and orexin directly activates VTA dopaminergic neurons (Nakamura et al., 2000; Korotkova et al., 2003). Icv orexin-induced hyperlocomotion and stereotypy were blocked by a dopamine receptor antagonist (Nakamura et al., 2000). Moreover, icv or local VTA infusion of orexin was shown to reinstate drug-seeking or food-seeking behavior in rodents (Boutrel et al., 2005; Harris et al., 2005).

**Figure 4:**
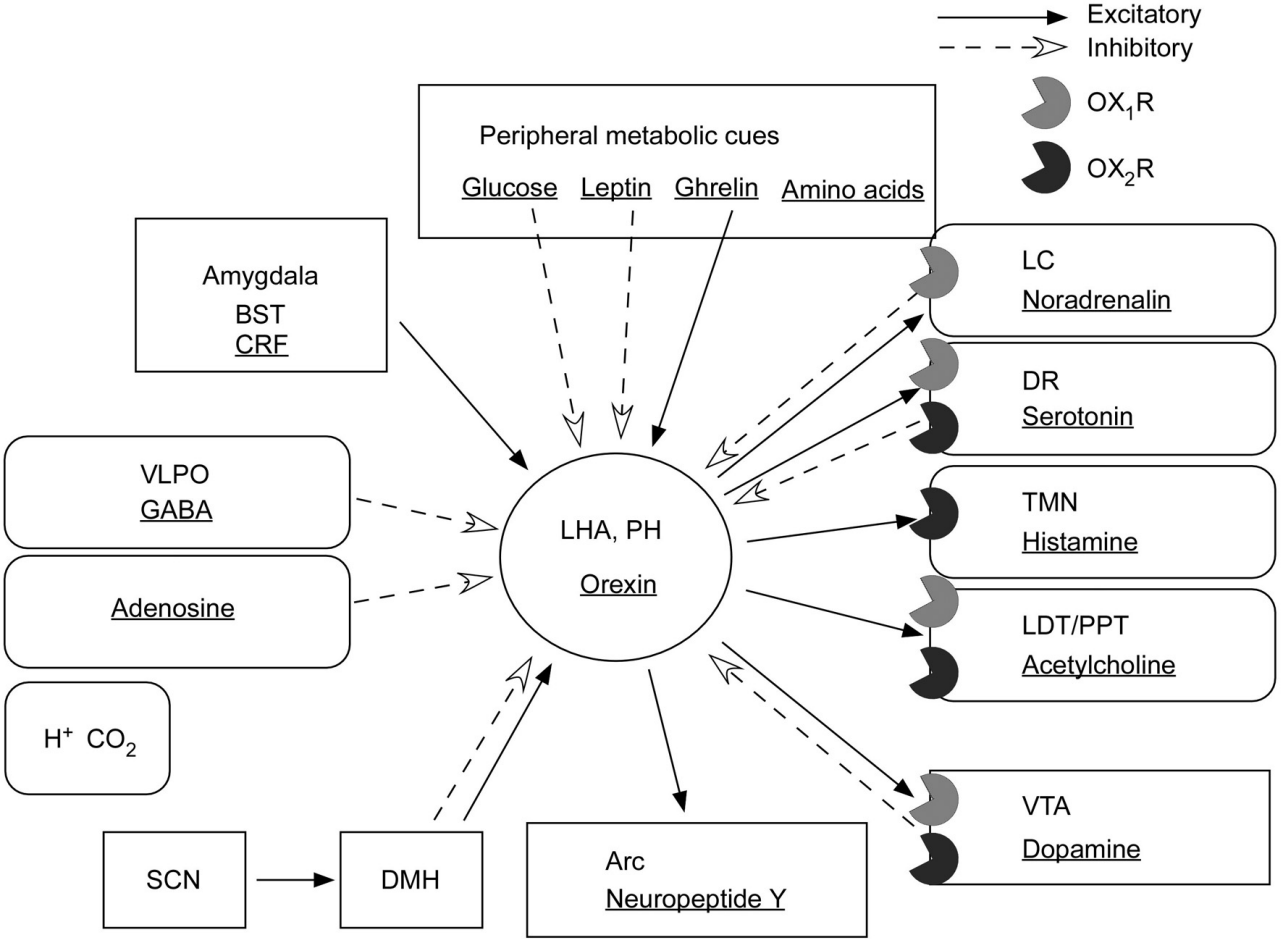
**Input and output of orexin neurons at interface of sleep, stress, reward, and energy homeostasis.** Orexin neurons in the lateral hypothalamic area (LHA) and posterior hypothalamus (PH) are placed to provide a link between the limbic system, energy homeostasis, the brain stem, and other systems. Arrows show excitatory projections and broken arrows inhibitory projections. Gray semicircles indicate OX_1_R and black semicircles indicate OX_2_R. Neurotransmitters/modulators are underlined. LC, DR, and TMN are wake-active regions, VLPO is sleep-active region, and LDT/PPT is REM-active region. Orexin neurons promote wakefulness through monoaminergic nuclei that are wake-active. Stimulation of dopaminergic centers by orexins modulates reward systems (VTA). Peripheral metabolic signals influence orexin neuronal activity to coordinate arousal and energy homeostasis. Stimulation of neuropeptide Y neurons by orexin increases food intake. The SCN, the central body clock, sends input to orexin neurons via the DMH. Input from the limbic system (amygdala and BST) might be important to regulate the activity of orexin neurons upon emotional stimuli to evoke emotional arousal or fear-related responses. Abbreviations: BST, bed nucleus of the stria terminalis; VLPO, ventrolateral preoptic area; LC, locus ceruleus; DR, dorsal raphe; TMN, tuberomammillary nucleus; LDT, laterodorsal tegmental nucleus; PPT, pedunculopontine tegmental nucleus; VTA, ventral tegmental area; SCN, suprachiasmatic nucleus; DMH, dorsomedial hypothalamus; Arc, arcuate nucleus.

On the contrary, orexin neurons receive projections from the VTA, NAc, and LS—regions involved in reward systems (Yoshida et al., 2006). Consistently, dopamine has an inhibitory influence on food intake and reward pathways when injected into the LHA/PFA (Yang et al., 1997). These reciprocal interactions might constitute regulatory mechanisms of reward systems (Figure 4).

Furthermore, many reports suggest a critical role of orexin signaling in neural plastic effects at glutamatergic synapses in the VTA. *In vivo* administration of an OX_1_R antagonist, SB334867, inhibited cocaine- or high fat-induced potentiation of excitatory currents in VTA dopaminergic neurons (Borgland et al., 2006, 2009). Orexin A input to the VTA potentiates N-methyl-D-aspartate receptor (NMDAR)-mediated neurotransmission via PLC/PKC-dependent recruitment of NMDA receptors in dopamine neuron synapses (Borgland et al., 2006). Moreover, orexin B increased presynaptic glutamate release in addition to postsynaptic potentiation of NMDA receptors in the VTA (Borgland et al., 2008). These findings suggest roles of orexin in the mechanisms of reward systems and drug addiction (Figure 4).

#### Motivation for positive reinforcement

Behavioral studies have suggested that orexin neurons play an important role in motivation, feeding, and a variety of reward-seeking behaviors. For example, orexins are involved in self-administration behavior. Under fixed ratio schedules, SB334867 reduced self-administration of nicotine, alcohol, high-fat pellets and sucrose in food-restricted rats, and heroin (Hollander et al., 2008; Nair et al., 2008; Cason et al., 2010; Jupp et al., 2011; Smith and Aston-Jones, 2012). Under a progressive schedule that requires the animal to lever-press progressively more times to obtain a reinforcer, SB-334867 also reduced the motivation to self-administer sucrose in food-sated, but not food-restricted rats, and cocaine (Borgland et al., 2009; Espana et al., 2010). These results show that OX_1_R signaling is important for motivation for a variety of highly salient, positive reinforcement. Orexin is also involved in reward-based feeding. Furthermore, orexin is also involved in cue-induced reinstatement of seeking for rewards. SB-334867 blocked foot shock-induced reinstatement of cocaine-seeking behavior (Boutrel et al., 2005; Smith and Aston-Jones, 2012) and an olfactory cue-induced reinstatement of alcohol-seeking behavior (Jupp et al., 2011). These findings suggest that orexin serves to motivate the animal to engage in goal-directed behavior interacting with reward systems.

## Conclusion

Orexin neurons provide crucial links between energy balance, emotion, reward systems, and arousal (Figure 4).

Symptoms of narcolepsy undoubtedly show that the orexin system plays highly important roles in regulating sleep/awake states and the maintenance of arousal by reciprocal interaction between orexin neurons and monoaminergic/cholinergic nuclei in the brain. The orexin system is also related to the limbic system, which regulates emotional responses, the reward system in the VTA, and hypothalamic mechanisms that regulate feeding behavior. These findings suggest that the orexin system senses the body's external and internal environments to regulate the state of arousal. Recently, a pharmaceutical company has stated that it plans to apply in 2012 for U.S. regulatory approval to market a dual orexin receptor antagonist for insomnia. Future research on the effect of orexin receptor antagonists in humans might shed light on the role of the orexin system more distinctly.

### Conflict of interest statement

The authors declare that the research was conducted in the absence of any commercial or financial relationships that could be construed as a potential conflict of interest.
